# Just try it: Preliminary results of a six‐month therapy for borderline personality disorder

**DOI:** 10.1002/pmh.1555

**Published:** 2022-06-25

**Authors:** Sylvia Martin, Jonathan Del‐Monte

**Affiliations:** ^1^ Center for Research and Bioethics Uppsala University Uppsala Sweden; ^2^ Psycho‐TCCE Private Practice Nîmes France; ^3^ Clinical Psychology Department Nîmes University Nîmes France; ^4^ LPS laboratory Aix‐Marseille University Aix‐en‐Provence France

## Abstract

Borderline Personality Disorder (BPD) is labeled as a personality disorder with impulsivity issues regarding relationships, risky behavior, and emotional dysregulation. This personality disorder is still considered to be hard‐to‐treat condition even though responses to treatment have improved significantly. In this field, Dialectical Behavioral Therapy is recognized as one of the most efficient therapeutical approaches, and some versions adapted from this gold‐standard treatment proved their effectiveness in clinical settings. We tested the 6‐month cognitive behavioral therapy (CBT) protocol ECCCLORE, composed of three modules, respectively, working on emotional regulation, distress tolerance, and relationships—in a French context to compare its effectiveness to Treatment As Usual. We recruited 56 patients with a BPD diagnosis in a day‐care unit, 34 receiving ECCCLORE and 22 receiving TAU. We assessed BPD traits, impulsivity, aggressiveness, suicidal risk, and hopelessness. Our results revealed the effectiveness of the 6‐month ECCCLORE protocol to decrease BPD symptoms and associated clinical dimensions. We also noted the persistence of its effect after a 1‐year follow‐up. Shortened CBT treatment protocols tend to prove their efficiency for symptom reduction in BPD. Further research is needed to replicate these results.

## INTRODUCTION

Borderline Personality Disorder (BPD) comes from Cluster B in the Diagnostic and Statistical Manual of Mental Disorders 5th version (DSM‐5) classification of personality disorders. BPD prevalence is between 0.5% and 5.9% in the general population. It used to be considered as one of the most prevalent and impactful PD in psychiatric population: 10% of psychiatric outpatients and 15% to 25% of hospitalized patients (Leichsenring et al., 2011). Even if the results of these prevalence studies can be disputed depending on potential biases (sampling, methods, country, and data collection techniques), latest assessments still reveal prevalence rates vary from 0.7% to 1.4% (Trull et al., 2010). BPD is still a frequent PD diagnosed in psychological care as it is a combination of interpersonal dysregulation, impulsivity, and emotional dysregulation. Impulsivity appears as one of the most significant symptoms, and numerous studies have shown its link with suicide risk (Cyders et al., 2009; Cyders et al., 2007; Lynam et al., 2011; Peters et al., 2017; Anestis & Joiner 2011; Slotema et al., 2017).

BPD's gold standard treatment is Dialectical Behavioral Therapy (DBT). This treatment was conceived in 1993 by Linehan et al. Several studies demonstrated DBT's effectiveness for suicidal and self‐injurious behavior reduction, and emotional regulation improvement (Linehan et al., 2006, Panos et al., 2014; Mehlum et al., 2014; Van den Bosch et al., 2005). Nevertheless, this protocol is demanding for both patients and caregivers, as well as health‐care professionals as it lasts for at least 1 year. Additionally, it requires the implementation of phone‐based support services and intensive therapist training programs and supervision.

Therapists have started to test shortened versions of DBT all around the world. In 2017, Mc Main et al. demonstrated the effectiveness of a 20‐week DBT adaptation leading to hospital services' use reduction (Mitchell et al., 2019; Muhomba et al., 2017). Inline, Sleuwaegen et al. (2018) showed that a 3‐month program significantly improves symptomatology promoting patient adherence (Barnicot et al., 2011). In 2012, Soler et al. studied the effectiveness of one DBT module, which, on its own, reduced the impulsivity level. Dialectical behavioral therapy, in its brief format, has begun proving its effectiveness via randomized controlled trials (RCT) settings (McMain et al., 2009; McMain et al., 2017), but to our knowledge, no French DBT adaptation of the short version has been evaluated as of yet. Cognitive behavioral therapy (CBT) manualized therapies are still underrepresented in France. Treatment duration is proven to be adaptable, but the effectiveness of DBT could lie in the specific skills and close support that is given to the patient.

A review by Links et al. (2017), based on 184 studies, demonstrated that the intensive nature of psychotherapies was not predictive of the results. Assessing the difference in lengths and the severity of symptoms, they conclude that less extensive protocol is effective even for severe patients. The results in the aforementioned review made it possible to consider a shorter timeframe for DBT programs.

In 2018, Oud et al. completed an analysis of 20 studies (1375 patients) showing the effectiveness of “specialized” psychotherapies challenging the DBT hegemony (Mentalization Based Therapy, Transference Focused Therapy, and Schema Focused Therapy. Other studies prove the effectiveness of mindfulness‐based programs on BPD symptomatology (Wupperman et al., 2008; Soler et al., 2012; Welch et al., 2006). This specific method reduces general impulsivity (Peters et al., 2011; Soler et al., 2016) and its emotional correlates (Wupperman et al., 2009; Carmona I Carmona i Farrés et al., 2019; Perroud et al., 2012). These numerous evidences of effectiveness in psychotherapeutic interventions widened the field's possibilities for BPD psychotherapy novelties, and the authors have expressed their hope for novel protocols targeting specific symptoms such as impulsivity (Oud et al., 2018).

Based on these facts, we had created an adapted protocol for impulsivity and suicidal risk reduction for BPD, and we will be specifically looking at urgency dimensions, notably Negative Urgency, as they tend to play a role in BPD symptomatology (Howard & Khalifa, 2016; Martin et al., 2019; Peters et al., 2017; Taherifard et al., 2015).

This study aims to measure the BPD‐traits evolution and clinical measure outcomes before, during, and after the ECCCLORE protocol. ECCCLORE is a 6‐month psychotherapeutic protocol for BPD. This program focuses on emotional regulation, crisis management, and relational issues. We will hereby examine the BPD symptomatology and any associated clinical measure decrease. We aim to prove that ECCCLORE will be more effective than TAU and will have lasting effects.

## METHODS

Thirty‐four women diagnosed with BPD were recruited (mean age total = 49.16 years SD = 9.197; meantime from first diagnosis = 35.94 months SD = 59.21). We recruited 22 BPD patients that received Treatment as Usual (TAU) in an out‐care unit for at least the same amount of time (mean age TAU = 45.53 SD = 8.89; meantime from the first diagnosis = 23.09 months SD = 58.54). The mean age difference is statistically comparable as no significant differences exists in the results of the Mann–Whitney *U* comparison test. After 1 year, we collected data from the 27 participants (7 from TAU and 20 from ECCCLORE). According to the current treating psychiatrist, all participants met the DSM‐5 diagnosis for BPD (based on Structured Clinical Interview for Axis II Personality Disorders II criteria), presented no suicidal risk, and were in a stable phase. The researchers recruit all individuals with BPD from a university hospital and a psychiatric clinic with a day‐care unit. Exclusion criteria for both groups were (a) known neurological disease, (b) developmental disability, (c) current substance use disorder, and (d) current psychotic disorder. All participants were proficient in French and had no disabilities that could impair their understanding of the questionnaires. The participants gave written consent to participate in this experiment following Helsinki's ethics recommendations. A structured interview with the psychologist and confirmation from the treating psychiatrist established the patient's capacity to provide informed consent. Data collection was initially made via paper and pencil records but was moved to online data collection for post‐treatment.

Program ECCCLORE©: Training in Cognitive and Behavioral Skills Related to Emotional Observation and Regulation. The protocol is composed of 24 sessions led by 2 clinical psychologists specialized in Cognitive‐Behavioral and Emotional Therapy for 6 months. The protocol consisted of a weekly 3‐h group session and an individual interview with one of the two psychologists for 30 min every week.

**FIGURE 1:**
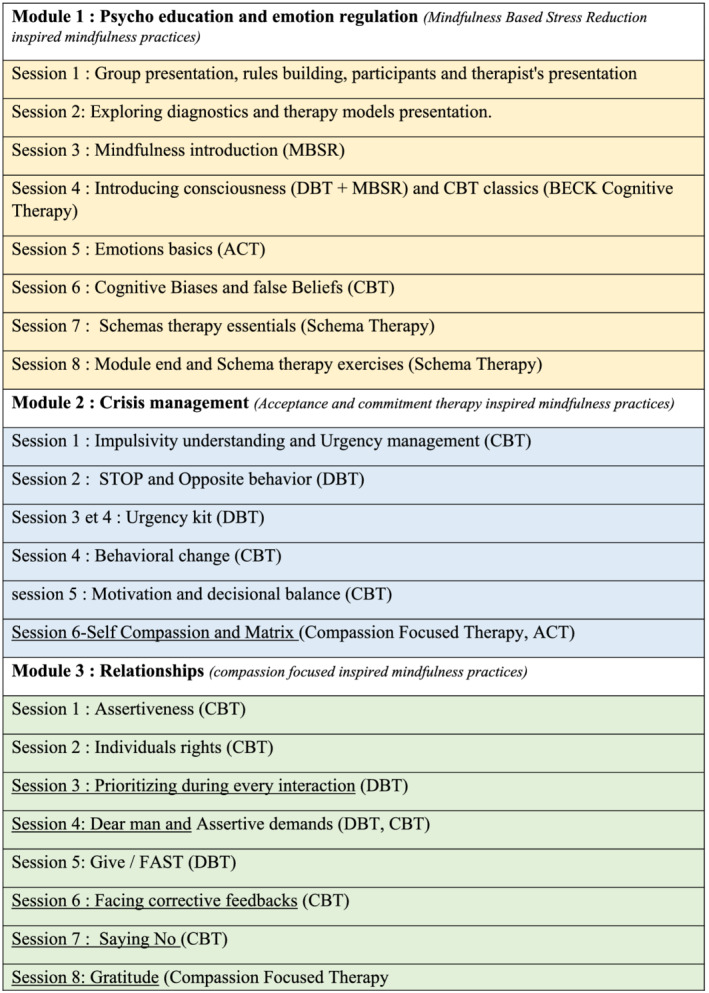
Treatment description

ECCCLORE group is three modules' based (See Figure 1).

*The First Module* (eight sessions) presents diagnosis items, teaches emotion recognition, enhances sensation recognition, and uses cognitive skills to increase “Enlightened Consciousness.” We teach mindfulness basics (only one sensory modality per session). This module builds up awareness of the disorder.

*The Second Module* (eight sessions) works on impulsivity issues. We use urgency management skills, motivation, and decision making. We implement Acceptance and Commitment Therapy (ACT) tools (value clarification and matrix work).

*The Third Module* (eight sessions) works on interpersonal skills and builds up self‐assertion and non‐violent communication. It fully integrates ACT skills. We implement self‐compassion practices (Gilbert & Plata, 2013).

Figure 1 presents a detailed description of each session.

TAU treatment consists of general outpatient psychiatric care (support groups and occupational therapy groups for at least 3 h a week) along with regular meetings with a psychiatrist or individual counseling with other caregivers (nurses, art therapists, and physical therapists).

### Measures

#### Borderline personality questionnaire (BPQ)

We assessed the importance of personality traits using the BPQ French Version with a Cronbach's *α* at 0.93 (Bianchi et al., 2018). BPQ is an 80‐item true/false self‐report measure that assesses borderline personality traits. This structure offered nine different scores, each of which for a corresponding BPD trait (impulsivity, affective instability, abandonment, relationships, self‐image, suicide/self‐mutilation, emptiness, intense anger, and quasi‐psychotic states).

#### UPPS impulsive behavior scale ‐ short version (UPPS‐S)

We used the French translation of the scale (Billieux et al., 2021). UPPS‐S is a self‐report scale composed of 20 items assessing four factors of impulsivity: (a) urgency (negative and positive), (b) lack of premeditation, (c) lack of perseverance, and (d) sensation seeking. Positive urgency and negative urgency evaluated the level of impulsivity due to positive or negative emotions, respectively. Globally, this instrument has a right consistency (negative urgency's Cronbach's *α* = 0.78, positive urgency's Cronbach's *α* = 0.70, lack of premeditation's Cronbach's *α* = 0.79, lack of perseverance Cronbach's *α* = 0.84, and sensation seeking's Cronbach's *α* = 0.83).

#### The aggression questionnaire (AQ12)

Buss and Perry created the aggression questionnaire in 1992 to assess physical and verbal aggression, anger, and hostility. We used the French translation of Genoud and Zimmermann, 2009. Cronbach's *α* was computed and suggested that the internal consistency is acceptable for three ‐item subscales (0.81 for physical aggression, 0.73 for verbal aggression, 0.77 for anger, and 0.80 for hostility) and considerable for the whole questionnaire (0.85). The participants must answer each item using a 5‐point Likert‐type scale (1 = uncharacteristic of me, 5 = very characteristic of me). The obtained score corresponds to the patient's aggression level through a global unidimensional measure.

#### Hopelessness scale (H)

The Hopelessness scale from Beck et al. (1974) was translated into French by Bouvard et al. in 1992. The scale has good reliability (test–retest, *r* = 0.81) and good internal consistency (Cronbach's *α* = 0.97) for depressive subjects and for control subjects (Cronbach's *α* = 0.79). The scale aims to evaluate pessimism and cognitive beliefs about the future and reflects the suicidal intentions indirectly. Items are completed via a binary quotation “true/false.” The total score varies from 0 to 20.

#### Suicidal behaviors (SBQR)

Osman et al. (2001) created this questionnaire assessing suicidal behaviors. Suicidal Behavior Questionnaire‐Revised (SBQ‐R) is one of the only tools asking about future anticipation of suicidal thoughts/behaviors, both in the past and in the present moment. It also includes questions about lifetime suicidal ideation, plans to commit suicide, and actual attempts. Shakeri et al. (2015) directed it to a psychiatric population. A total score of 7 or higher in the general population and a total score of 8 or higher in patients with psychiatric disorders indicate a significant risk of suicidal behavior, respectively. We used the French validation from Potard et al. (2014) (Cronbach's *α* was 0.97).

We computed non‐parametric tests using the Statistical Package for Social Sciences (SPSS) 20.0. as the samples were not normally distributed. The level of significance was set at *p* < 0.05. Mann–Whitney's *U* and Wilcoxon tests were used to explore the differences between the scale scores.

## RESULTS

The mean of measured “mean time from the first BPD diagnosis” stood at 23.67 months (SD = 45.66), and the mean “medications taken per day number” was 3 (SD = 1.76). Table 1 reports all clinical scale means.

**TABLE 1:** Mean and SD table according to the treatment stage

	Pre		Post 1		Post 2		Post 3 ECCCLORE		TAU	
	Mean	SD	Mean	SD	Mean	SD	Mean	SD	Mean	SD
Negative urgency	13.21	3.01	12.1	2.97	10.76	2.32	10.18	2.77	12.41	3.43
Positive urgency	12.76	3.46	11.9	2.88	10.71	2.39	11.23	2.40	12.55	3.18
Lack of premeditation	9.24	4.11	9.18	3.14	8.19	2.25	9.09	3	9.32	3.78
Lack of perseverance	8.88	4.14	8.18	2.59	9.14	3.55	0.45	3.93	8.73	4.10
Sensation seeking	10.35	4.70	10.1	3.05	9.71	3.33	10.64	2.93	10.50	4.20
UPPS	54.44	8.94	51.5	8.2	48.52	8.328	50.59	9.08	53.50	1.03
AQ12	45.06	11.26	42.8	10	34.55	10.14	35	10.13	41.76	14.0
SBQr	11.58	4.58	8.14	7.15	6.1	5.69	8.27	5.767	10	3.49
Impulsivity	3.88	2.16	3.68	1.94	2.59	1.84	3.32	1.729	3.50	1.97
Affect instability	7.66	1.97	7.36	2.59	6.77	2.94	6.41	2.72	6.95	2.43
Abandon	7.09	1.90	6.77	2.2	5.36	2.12	5	2.35	5.32	2.37
Relations	5.72	2.37	5,82	2.26	5.18	2.38	3.91	2.724	4.68	2.69
Self‐image	6.44	1.45	6.09	2.27	5.32	2.31	5.32	1.96	5.73	2.00
Suicide self‐mutilation	4.25	2.50	4.68	2.53	3.77	2.40	2.59	2.82	3.59	2.42
Emptiness	8.16	2.01	7.68	2.51	6.5	2.89	5.91	2.84	6.82	2.75
Intense anger	6.81	2.36	5.82	2.36	4.27	3.04	4.59	2.97	5.55	3.20
Psychotic	3.28	2.03	2.77	1.69	2.05	1.49	1.73	1.35	2.95	2.23
BPQTot	53.28	10.53	50.7	9.71	41.82	1.,2	38.77	13.22	45.09	12.65
H	12.84	4.36	10.5	5.15	8.09	5.68	9.14	5.15	10.33	5.72

*Note*: BPQTot: Borderline Personality Questionnaire Total score, H: Hopelessness.

**FIGURE 2:**
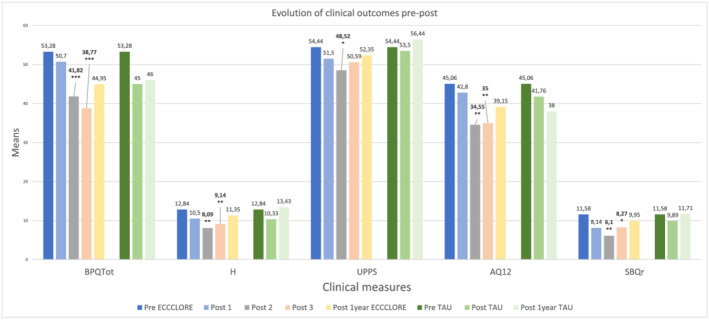
Evolution of clinical measures means and significant changes across pre‐post and follow‐up stages. Notes: BPQTot = Borderline Personality Questionnaire total score; H = Beck Hopelessness's score; UPPS = Impulsive behavior scale score; AQ12 = aggression level; SBQr = suicidal risk; * = *p* < 0.5;** = *p* < .05;*** = *p* < 0.01

We ran a Mann–Whitney's *U* test analysis to compare the mean evolution before, during, and after ECCCLORE, as well as before and after TAU. After the first module, not all dimensions significantly decreased. After the last module, most dimensions diminished significantly (see Table 2). Figure 2 offers a view of the mean evolution for UPPS, BPQ, AQ12, SBQr, and H scores. Wilcoxon analysis for intragroup evolution is presented in Table 3.

**TABLE 2:** Mean comparison

	Pre‐post 3 ECCCLORE			Pre‐post TAU			Pre‐post 1 ECCLORE			Pre‐post 2 ECCLORE		
	*U* de Mann–Whitney	Sig.	Cohen's *d*	*U* de Mann–Whitney	Sig.	Cohen's *d*	*U* de Mann–Whitney	Sig.	Cohen's *d*	*U* de Mann–Whitney	Sig.	Cohen's *d*
Negative urgency	174	**0.001** ***	0.104	22.5	0.835	0.024	320.5	0.363	0.513	182	**0.002** ***	0.091
Positive urgency	274	0.091	0.051	213	0.658	0.006	343.5	0.606	0.470	228	**0.024** *	0.083
Lack of premeditation	347	0.649	0.004	213.5	0.669	0.002	341.5	0.583	0.002	276	0.157	0.036
Lack of perseverance	361.5	0.833	0.020	213	0.658	0.005	314	0.312	0.203	351.5	0.924	0.010
Sensation seeking	352.5	0.717	0.007	218.5	0.76	0.003	374	1	0.280	339	0.754	0.0157
Impulsivity	312	0.474	0.028	215	0.694	0.018	340	0.83	0.013	241	**0.048** *	0.064
Affect instability	252.5	**0.075**	.088	201.5	0.466	0.031	347	0.929	0.021	307	0.422	0.035
Abandon	176	**0.002** **	0.518	143	**0.031** *	0.082	330.5	0.701	0.024	194	**0.005** **	0.086
Relations	211.5	**0.012** *	0.070	165.5	0.106	0.040	349	0.957	0.006	302	0.37	0.023
Self‐image	238	**0.041** *	0.064	201.5	0.461	0.040	350	0.971	0.033	259.5	0.097	0.058
Suicide and self‐mutilation	235.5	**0.037** *	0.062	172	0.146	0.026	308.5	0.436	0.024	306	0.412	0.019
Emptiness	165	**0.001** ***	0.091	186.5	0.272	0.055	314	0.493	0.033	222.5	**0.021** *	0.301
Intense anger	197.5	**0.006** **	0.0821	215.5	0.703	0.044	256	0.088	0.059	181	**0.002** ***	0.093
Psychotic states	191.5	**0.004** ***	0.089	229.5	0.97	0.015	302	0.372	0.035	228.5	**0.028** *	0.069

*Notes*: BPQTot, Borderline personality questionnaire Total score; H, Hopelessness.

* *p* < 0.05

** *p* < 0.005

*** *p* < 0.001

**TABLE 3:** Wilcoxon test for global intra group changes

		Post 1‐post 2	Post 1‐post 3	Post 2‐post 3
H	*Z*	1.603	0.374	0.430
	*p*	0.109	0.708	0.667
UPPS	*Z*	0.878	0.400	0.800
	*p*	0.380	0.689	0.424
BPQ tot	*Z*	2.632	2.573	0.568
	*p*	**0.008** *	**0.010** *	0.570
SBQR	*Z*	1.164	0.226	1.200
	*p*	0.245	0.821	0.230
AQ12	*Z*	2.590	2.148	0.146
	*p*	**0.010** *	**0.032** *	0.884

* *p* < 0.05.

After the second module, scores significantly decreased: Negative Urgency (*p* = 0.002, Cohen's *d* = 0.091), Positive Urgency (*p* = 0.024, Cohen's *d* = 0.083); UPPS global score (*p* = 0.018, Cohen's *d* = 0.068); AQ12 (*p* = 0.003, Cohen's *d* = 0.131); SBQ‐r (*p* = 0.002, Cohen's *d* = 0.212); Impulsivity (*p* = 0.048, Cohen's *d* = 0.064); Abandonment sensitivity (*p* = 0.005, Cohen's *d* = 0.086); Emptiness (*p* = 0.021, Cohen's *d* = 0.301); Psychotic states (*p* = 0.028, Cohen's *d* = 0.069); and BPQ total score (*p* = 0.001, Cohen's *d* = 0.154).

Our results showed an evolution of the impulsivity dimension throughout the program. Negative Urgency significantly decreased (*p* = 0.001, Cohen's *d* = 0.105), while Positive Urgency only tended to decrease (*p* = 0.091, Cohen's *d* = 0.051), weighing down the whole impulsivity level (Table 4).

**TABLE 4:** Means for post treatments after 1 year

	Post 1 year TAU		Post 1 YearECCCLORE	
	Mean	SD	Mean	SD
Negative urgency	14.95	5.266	16	5.447
Positive urgency	10.8	4.262	12	6.429
Lack of premeditation	9.5	4.431	12.29	4.192
Lack of perseverance	7.6	2.521	8.86	3.891
Sensation seeking	9.5	2.763	7.29	2.498
AQ12	39.15	10.927	38	14.56
SBQr	9.95	3.591	11.71	3.946
Impulsivity	3.95	2.235	3	2.309
Affect instability	6.95	2.46	8.14	1.952
Abandon	5.3	2.273	4.14	1.864
Relationships	4.25	2.807	3.71	3.302
Self‐image	5.9	2.594	6.43	2.637
Suicide	3.85	2.54	5.71	1.496
Emptiness	6.8	2.628	7.57	3.735
Intense anger	4.55	2.929	4	3.559
Psychotic	3.4	1.759	3.29	2.628
BPQTot	44.95	12.584	46	15.674
H	11.35	5.441	13.43	6.079

### After 1‐year follow‐up results

Means comparisons are presented in Table 5.

**TABLE 5:** Mean comparisons after 1 year follow‐up

	Follow‐up TAU/follow‐up ECCCLORE		Post TAU/follow‐up TAU		Post ECCCLORE/follow‐up ECCCLORE		Pre/follow‐up ECCCLORE		Pre/follow‐up TAU	
	Mann–Whitney's *U*	p	Mann–Whitney's *U*	*p*	Mann–Whitney's *U*	*p*	Mann–Whitney's *U*	*p*	Mann–Whitney's *U*	*p*
Negative urgency	61.5	0.637	43	0.103	95.5	**0.002** ***	243	0.08	81.5	0.189
Positive urgency	62.5	0.676	69	0.81	197	0.559	232	0.052	103.5	0.59
Lack of premeditation	44	0.148	45	0.128	218.5	0.97	318	0.692	75	0.125
Lack of perseverance	61	0.615	72.5	0.957	163	0.148	260	0.15	110.5	0.767
Sensation seeking	39	0.085	41	0.083	173.5	0.238	314	0.639	73	0.109
AQ12	67	0.868	65	0.782	179.5	0.307	234	0.105	82	0.272
SBQr	50.5	0.278	46.5	0.244	161.5	0.14	212.5	0.29	87	0.859
Impulsivity	54.5	0.384	62.5	0.555	186.5	0.392	315	0.924	86.5	0.344
Affect instability	50	0.261	53.5	0.282	198	0.576	269.5	0.336	94.5	0.515
Abandon	49.5	0.251	55	0.321	202.5	0.657	175	**0.006** **	29.5	**0.002** **
Relations	62.5	0.675	59	0.435	205.5	0.713	222.5	**0.063**	72	0.134
Self‐image	58.5	0.504	54	0.287	167	0.175	314	0.907	94.5	0.507
Suicide	39.5	0.085	36.5	**0.046** *	162	0.134	293.5	0.612	74	0.157
Emptiness	48	0.215	58	0.396	170.5	0.207	210	**0.034** *	103.5	0.749
Intense anger	62.5	0.676	55	0.324	216.5	0.929	175	**0.006** **	57	0.042
Psychotic	61	0.611	67	0.726	93	**0.000** ***	309	0.834	109.5	0.926
BPQTot	64	0.739	60	0.473	161	0.137	192	**0.016** *	90	0.42
H	52	0.318	49	0.244	165	0.165	270	0.345	95.5	0.545

* *p* < 0.05

** *p* < 0.005

*** *p* < 0.001.

Concerning TAU results, the only difference 1 year after the end of care was on the suicide/self‐mutilation dimension of the BPQ scale (Mann–Whitney's *U* = 36.2, *p* = 0.046). For the ECCLORE post‐treatment and post‐1‐year means comparisons, differences laid in Negative Urgency (Mann–Whitney's *U* = 0.95, *p* = 0.002) and Psychotic trait (Mann–Whitney's *U* = 0.93, *p* = 0.001). More precisely, comparing pre‐treatment scores to the ones we collected for the 1 year follow‐up in the ECCCLORE sample, differences laid in the Negative Urgency (Mann–Whitney's *U* = 0.243, *p* = 0.08) Positive Urgency (Mann–Whitney's *U* = 0.232, *p* = 0.052) Abandonment (Mann–Whitney's *U* = 175, *p* = 0.006), Relationships (Mann–Whitney's *U* = 222.5, *p* = 0.063) Emptiness (Mann–Whitney's *U* = 210, *p* = 0.034) Intense Anger (Mann–Whitney's *U* = 175, *p* = 0.006) and Total BPQ (Mann–Whitney's *U* = 192, *p* = 0.016) (see Table 5).

## DISCUSSION

The results show that ECCCLORE led to a significant reduction in general symptomatology, aggression, hopelessness, impulsivity, and suicidal risk. These results are significantly better than TAU.

Our results are coherent with similar settings, even though shorter protocols, like Laporte et al. in (2017), which implemented a short treatment (12 weeks) based on psychoeducation, and group processes showed benefits on clinical measures.

Another outcome is that results are significant after the second module. This is consistent with the literature showing 3 months based on DBT protocols' effectiveness (Soler et al., 2009). ECCCLORE's results call for treatment duration adaptations with different timeframes depending on the clinical setting (for a review, see Bloom et al., 2012; Soler et al., 2009; McMain et al., 2017, Fleischhaker et al., 2011; Yen et al., 2009). In 2015, Paris's review reported sustainable behavior changes after 6 months, so further research needs to compare different treatment options from DBT to CBT treatments.

This difference can lie in the intensity of these shortened protocols. In their meta‐analysis, Oud et al. in 2018 referenced the lengths and time per week spent with the patient in different RCTs, comparing DBT with TAU or other therapies. Globally, for the outpatient, the time spent varied from 1 h to 6.5 h per week. In a study comparable to ECCCLORE (26 weeks); one treatment dedicated 5.9 h per week of care to the patient, but for hospitalized patients (Bohus et al., 2013), another one dedicated 3 h weekly to individual sessions (Koons et al., 2001). Koons et al. (2001) reported a significant effect at mid‐treatment for depression, anxiety, and anger (at 13 weeks). At the end of treatment, changes occurred in suicidal measures, hopelessness, anger, depression, and BPD criteria. With ECCCLORE, we get results after two modules, which could also be due to this difference as our time of 3.5 h per week was differently distributed: 3 h of group sessions and 30 min of individual therapy. Further research is needed to examine different timeframes considering both clinical and practical aspects (Pasieczny & Connor, 2011).

For clinical improvements, our results show that Impulsivity decreased due to the Negative Urgency decrease. This result encourages us in focusing on more specific exercises targeting specific impulsivity dimensions. For example, Negative Urgency‐centered interventions could focus on emotional regulation, distress tolerance, and interpersonal effectiveness (Zapolski et al., 2010). The effect on Negative Urgency in our results aligns with Zapolski et al.'s recommendation that Negative Urgency should be addressed via emotional regulation, distress tolerance, and interpersonal effectiveness, but also soothing techniques, and effective communication. For Positive Urgency, they recommend using targeted techniques to help the patient savor success, and some which encourage positive moods. We can perceive how ECCCLORE can then be well suited for Urgency work. This can be due to Negative Urgency skills being taught throughout the protocol, whereas most of Positive Urgency‐oriented skills are only offered by the end of the protocol. This explains why results show little effect on Positive Urgency. Moreover, as recent research development has started to question the validity of Urgency categorization stating that positive and negative forms could be the same impulsivity component (Billieux et al., 2021), further research must clarify the need to consider new constructs addressing emotion‐based impulsivity. Howard and Khaliga in 2016 shouldered the impact of Urgency as a core feature of PD, proving that its recognition as a predictive factor of PD severity and its relation to violence may still be relevant to understand BPD's emotion‐related impulsivity dynamics.

The 1‐year follow‐up ECCCLORE participants observed decreased symptomatology. Similarly, this is consistent with DBT, in which results are found after a 1‐year follow‐up (Fleischhaker et al., 2011). No significant differences were found between TAU and ECCCLORE scores post‐1‐year follow‐up stage, but these results must be carefully considered due to the possible impact of the pandemic on the French population which had a general increase in anxiety and depressive symptoms at the time (Chaix et al., 2020; Peretti‐Watel et al., 2020; Weill et al., 2020).

This study has several limitations. The first is its general sample size and its gender bias (this preliminary study recruited only women). Considering that it is a preliminary study, we have a second limitation with no efficient follow‐up data at this stage. A third limitation comes from the small sample size for post‐1‐year evaluation as it was only possible to recruit seven TAU participants. Moreover, the special conditions of the global pandemic and several months of isolation in France could have created several biases. A final limitation is that we did not have medication data collection from post‐treatment and post‐1‐year treatment.

Further studies are needed to realize a comparison between psychotherapeutic approaches. This is true even more so now, as some research has started to question the effect of renowned DBT protocols (Cristea et al., 2017; Links et al., 2017; Oud et al., 2018). Several meta‐analyses in recent years (Finch et al., 2019; McLaughlin et al., 2019) have proven that group psychotherapy has a large effect on symptom reduction and a moderate effect on suicidality/parasuicidality. However, they only found small to medium effects which are in favor of group treatment for other clinical issues that may be relevant to treat in a BPD population where comorbidity is frequent (McLaughlin et al., 2019). Another interesting finding, from Finch et al.'s meta‐analysis on TAU's effectiveness, was that comparative treatments used in RCTs had small to moderate effects on BPD symptoms and showed small effects on other clinical dimensions. This puts in question the usefulness of some elements from TAU that could help develop further therapies that include “general psychiatric care” elements. Adding to these observations, specific results proved that integration is not always effective, and the integration of CBT elements to psychodynamic care does not sufficiently improve therapeutical outcomes (Goldman et al., 2018). There is a further need to address the specific leverage that different CBT techniques could offer for BPD integrative psychotherapy settings.

## CONCLUSION

Implementing a new, shorter protocol based on classic CBT could encourage researchers and practitioners to work with BPD patients. Treatment goals for impulsivity or aggression may lead to the development of more effective DBT program updates.

Further developments of ECCCLORE will include a more specific focus on Zapolski et al.'s (2010) recommendations on addressing specific sub‐dimension of impulsivity. They might need to consider that the most severe symptomatology predictor is still a moderator of outcomes from psychotherapies (Sahin et al., 2018). Recent studies continue exploring how to increase the impact of the therapeutic offer (Boritz et al., 2018). Further studies could consider the effect of psychotherapeutic approaches depending on suicidal history, BPD severity, and/or comorbid disorders like DBT protocols adapted to trauma (Harned & Korslund, 2015), or Bohus et al.'s (2013) DBT‐PTSD, as these specific treatments show greater effectiveness with no adverse effects.

## CONFLICT OF INTEREST

None.

## ETHICAL APPROVAL AND CONSENT

Every participant signed an informed consent form for publication and data use for research purposes.

## FUNDING INFORMATION

None.

## AUTHOR CONTRIBUTIONS

All authors made substantial contributions to the conception and design of the work.

## Data Availability

Data and materials will be available under reasonable demand to the corresponding author. A preliminary presentation has been made at two international congresses to raise interest and communicate around the first outcomes.
